# Impact of the Italian Society of Anatomic Pathology and Diagnostic Cytology Classification of Thyroid Nodules in the Treatment of Indeterminate Follicular Lesions: Five-Year Results at a Single Center

**DOI:** 10.1155/2020/7325260

**Published:** 2020-04-14

**Authors:** M. Pastoricchio, A. Cubisino, A. Lanzaro, M. Troian, F. Zanconati, S. Bernardi, B. Fabris, N. de Manzini, C. Dobrinja

**Affiliations:** ^1^Division of Clinical Surgery, Department of Medical, Surgical and Health Sciences, University of Trieste, Cattinara Teaching Hospital, Trieste 34149, Italy; ^2^Department of Medical, Surgical and Health Sciences, Università Degli Studi di Trieste, Cattinara Teaching Hospital, Strada di Fiume 447, 34149 Trieste, Italy; ^3^Endocrinology Unit—Azienda Sanitaria Universitaria Integrata Trieste, Cattinara Teaching Hospital, Strada di Fiume 447, 34149 Trieste, Italy

## Abstract

**Purpose:**

Aim of the study was to assess the impact of the Italian Society of Anatomic Pathology and Diagnostic Cytology (SIAPEC) classification of 2014, on the treatment of indeterminate thyroid lesions (TIR3).

**Methods:**

We retrospectively analyzed patients undergoing thyroid surgery for TIR3 lesions between 2013 and 2018, at the General Surgery Department of Trieste University Hospital. According to the SIAPEC classification, patients were divided into TIR3A and TIR3B groups. All patients treated before 2014 underwent surgical treatment, and surgical specimens were retrospectively classified after revision of fine-needle aspiration cytology. Starting 2014, TIR3A patients were treated only when symptomatic (i.e., coexistent bilateral thyroid goiter or growing TIR3A nodules), whereas TIR3B patients always received surgical treatment. Hemithyroidectomy (HT) was the procedure of choice. Total thyroidectomy (TT) was performed in case of concurrent bilateral goiter, autoimmune thyroid disease, and/or presence of BRAF and/or RAS mutation. Lastly, we analyzed the malignancy rate in the two groups.

**Results:**

29 TIR3A and 90 TIR3B patients were included in the study. HT was performed in 10 TIR3A patients and 37 TIR3B patients, respectively, with need for reoperation in 4 TIR3B (10.8%) patients due to histological findings of follicular thyroid carcinoma >1  cm. The malignancy rates were 17.2% in TIR3A and 31.1% in TIR3B, (*p* = 0.16). Predictability of malignancy was almost 89% in BRAF mutation and just 47% in RAS mutation.

**Conclusions:**

The new SIAPEC classification in association with biomolecular markers has improved diagnostic accuracy, patient selection, and clinical management of TIR3 lesions.

## 1. Introduction

Thyroid nodules have become a common finding in clinical practice, being detected in up to 67% of general population due to widespread use of sensitive imaging techniques [1–5]. Although most nodules are benign, thyroid cancer is detected in up to 5–15% of cases depending on several risk factors (e.g., age, sex, family history, and previous radiation exposure) [6–8]. The most common malignancy is differentiated thyroid cancer (DTC), which includes papillary and follicular histologies and represents the vast majority (>90%) of all thyroid cancers [9].

According to current guidelines, management is primarily based on morphologic classification of cytologic samples obtained by fine-needle aspiration (FNA), complemented by clinical and imaging findings, and increasingly supplemented by molecular markers test results [10–13]. Various classification schemes based on thyroid cytology have been proposed and their validity has been extensively demonstrated [10–13]. However, approximately 25% of all FNAs will fall into an indeterminate category, which represents a difficult challenge for clinicians since malignancy, although relatively low (20–30%), cannot be safely excluded [14–18]. FNA cytology is particularly unreliable in differentiating between benign and malignant follicular thyroid disease as the specific characteristics of aggressiveness (i.e., capsular and/or vascular invasion) can be established only after thorough histopathologic examination [15, 18]. As a result, most patients with indeterminate cytology at FNA biopsy will require diagnostic surgery, but only 20% of cases will turn out to be malignant, leading to more than 80% of unnecessary surgery excisions with associated risks and costs [14–18].

Classification systems have been trying to address the issue of reducing unneeded surgery without missing potentially malignant nodules by subdividing the indeterminate category into two different classes based on architectural features, grade of nuclear atypia, and relative risk of malignant occurrence (Table 1). In 2007, the United States National Cancer Institute (NCI) proposed the Bethesda System for Reporting Thyroid Cytology (BSRTC), in which indeterminate nodules were divided in class III or AUS/FLUS (atypia of undetermined significance or follicular lesion of undetermined significance) and class IV or FN/SFN (follicular neoplasms or suspicious for a follicular neoplasm), with different expected malignancy rates and management options [19]. Similarly, in 2009, the Royal College of Pathologists (RCPath) revised the British Thyroid Association (BTA) guidelines and divided the indeterminate THY3 category into THY3a (atypia/nondiagnostic) and THY3f (follicular lesion/suspect follicular neoplasia) [20]. More recently, the Italian Society of Anatomic Pathology and Diagnostic Cytology (SIAPEC) modified the previous classification of TIR3 indeterminate lesions by introducing two different subclasses, namely, TIR3A (low-risk indeterminate), for which clinical and radiological follow-up may be adequate, and TIR3B (high-risk indeterminate), for which surgery is always required [21, 22]. It has to be noted that, unlike the BSRTC and BTA-RCPath systems, the 2014 SIAPEC classification extended the TIR3B category to include those cases with mild or focal nuclear atypia suggestive of papillary carcinoma, which are expected to have a higher risk of malignancy [13, 21, 22]. Anyway, estimated rates of cancer are less than 10% for TIR3A and 15–30% for TIR3B, which are essentially similar to those reported by the BSRTC and BTA/RCPath classifications (i.e., 5–15% rates for the AUS/FLUS and THY3a categories and 15–30% for the FN/SFN and THY3f categories, respectively) [19, 20].

Several studies evaluating the BSRTC and BTA/RCPath classification systems have reported higher than expected malignancy rates [23, 24], whereas a more accurate estimate of the risk of malignancy for the indeterminate lesions have been observed with the adoption of the 2014 SIAPEC classification [25–28].

The aim of the present study was to assess the clinical impact of the 2014 SIAPEC classification on the management of indeterminate thyroid lesions, particularly with regard to malignancy rates and care strategies, including the role of surgery as a treatment option and the diagnostic impact of molecular markers in predicting malignancy.

## 2. Methods

We performed a retrospective analysis of all surgical procedures performed for thyroid disease at the General Surgery Department of Trieste University Hospital between January 2013 and January 2018. All patients with solitary thyroid nodules and preoperative cytological diagnosis of indeterminate follicular lesion were enrolled in the study and assigned to two independent groups, namely, TIR3A and TIR3B, according to the 2014 SIAPEC classification. For those treated before the introduction of the new system classification, subdivision was performed after retrospective revision of FNA cytology. Of these, the vast majority of patients fell into the TIR3B category.

Preoperative work-up of thyroid nodules consisted of full clinical examination and ultrasonography (US) of the neck, followed by FNA when nodules were >1 cm of diameter and/or presented US features of malignancy (e.g., solid composition, hypoechogenicity, irregular margins, microcalcifications, and cervical lymph node involvement). The US classification was obtained for all thyroid nodules according to the thyroid imaging reporting and data system (TIRADS) [10, 11, 29–31]. Based on US appearance, we distinguished two major categories: nodules presenting with a low to intermediate US risk of malignancy (TIRADS 3, 4a, and 4b) and those with a high risk of malignancy (TIRADS 4c and 5). TIR3A patients underwent clinical and instrumental follow-up, including neck US. The US appearance of the nodules and their initial size, in many cases, oriented the management strategy of these patients with consequent inclusion to surgery or to follow-up.

All FNAs were performed under US guidance by an experienced radiologist. Smears were either fixed in alcohol and stained by Papanicolaou stain or air dried and stained with Giemsa stain. Starting 2014, all TIR3B lesions and specific TIR3A nodules (i.e., those growing in size and those with US features of malignancy) are tested for BRAF mutation. From 2015, molecular testing includes RAS mutation assessment as well [32–35].

The material was obtained after digitization of the cytological specimen to define the gene structure for detecting the main and frequent mutations that are thyroid related. The molecular biology survey was performed on DNA extracted from cytological material aspirated by nodule of the thyroid gland and was conducted by real-time PCR technique after extraction with QIAamp DNA Mini Kit. The analysis detected the main mutations of codons 594, 600, and 601 as concerns BRAF and of codons 12, 13, 59, 61, 117, and 146 as concerns RAS (NRAS, KRAS, and HRAS). The quality of the laboratory test was submitted by the Associazione Italiana di Oncologia Medica (AIOM)-SIAPEC control. Particularly in TIR 3A, the mutational status of RAS gene provided us the most useful information to guide the management decision.

The molecular markers were investigated in all TIR 3B patients and in a fraction of TIR3A patients. The reason driving the decision to perform the molecular analysis just in some TIR3A nodules was to investigate if the presence of such mutations could justify the growing in size of these nodules, thus prompting the surgical treatment instead of the follow-up suggested in these patients. Moreover, the presence of such mutations was helpful in guiding the most appropriate surgical strategy.

Management options are based on FNA cytology, according to the 2014 SIAPEC classification and the American Thyroid Association (ATA) guidelines [10, 13, 22, 30]. Therefore, TIR3A patients underwent clinical and instrumental follow-up, consisting of serial medical evaluations performed by an endocrinologist, neck US, and laboratory testing. Surgery was considered only for symptomatic nodules, coexisting bilateral thyroid goiter, nodules growing in size and/or US features of malignancy. Hemithyroidectomy (HT) was the procedure of choice for solitary nodules, whereas total thyroidectomy (TT) was performed in case of bilateral thyroid goiter or in the presence of BRAF/RAS mutation, where available.

As far as TIR3B lesions are concerned, all patients underwent surgery. HT was the procedure of choice in case of intraparenchymal nodules with no known risk factors (i.e., negative BRAF/RAS status on FNA cytology, no history of previous head and neck irradiation, and no clinical nor radiological evidence of either nodal disease or distant metastases). TT was selectively proposed in the following cases: coexisting bilateral goiter, autoimmune thyroid disease, presence of BRAF/RAS mutation, prior head and neck irradiation, and family history of thyroid malignancies.

According to ATA guidelines [10, 30], in case of intraoperative finding of lymph nodes suspicious for metastasis, either unilateral or bilateral central neck dissection (CND) and/or lateral neck dissection (LND) were performed. Whenever final histopathology diagnosed malignancy in HT specimen, completion thyroidectomy was performed in the presence of one or more of the following: carcinoma >10  mm in size, multifocal disease with an overall sum of all lesions' diameters >10 mm, microscopic extrathyroid extension, and aggressive histologic features (i.e., tall-cell, columnar-cell, or diffuse sclerosing variants).

Histopathologic data (e.g. multifocality, aggressive features, extracapsular invasion, and lymph node metastases) were recorded for all patients. Noninvasive follicular thyroid neoplasms with papillary-like nuclear features (NIFTPs) were considered as nonmalignant, according to the reclassification introduced into the 2017 World Health Organization [36].

The malignancy rates were analyzed in both groups in order to evaluate whether the 2014 SIAPEC classification improved diagnostic accuracy and if its association with biomolecular markers could improve prediction of malignancy. Furthermore, histopathologic data were analyzed in order to determine whether completion thyroidectomy or total thyroidectomy were appropriate or should have been considered overtreatment.

### 2.1. Statistical Analysis

Categorical variables were assessed using Fisher's exact test or Chi-squared test, when appropriate. Continuous variables were evaluated by using Student's *t*-test or Mann–Whitney *U*-test, when appropriate. A *p* value of less than 0.05 was considered to be statistically significant. All statistical analyses were performed using GraphPad software.

## 3. Results

According to preoperative FNA cytology, a total of 119 patients underwent surgery for TIR3 lesions between January 2013 and January 2018. Of these, 29 cases had TIR3A nodules and 90 cases had TIR3B nodules. Population characteristics, including size (major diameter) and US appearance of the nodules, are summarized in Table 2.

In the TIR3A group, 10 patients underwent HT and 19 patients underwent TT. In the TIR3B group, 37 patients underwent HT and 53 patients underwent TT. Of those with positive molecular mutations, 2 TIR3A patients and 2 TIR3B patients preferred to be treated with HT despite accurate information upon potential risks and benefits of complete thyroidectomy. On the other side, TT was required by 4 patients although no thyroid goiter and/or known risk factors for malignancy were present.

Among TIR3A patients undergoing HT, none required completion thyroidectomy since final histological examination revealed benign nodules in 9 cases (including one case of NIFTP), whereas 1 patient presented with occult papillary thyroid microcarcinoma (PTMC) with no need for further surgery according to current guidelines [10]. Among TIR3A patients undergoing TT, malignancy was found in 4 patients, mostly in the form of PTMC (3 out of 4 cases, 75.0%). Unilateral CND was performed in 1 case due to intraoperative suspect of lymph node involvement, later confirmed at final histopathology. No case presented either extracapsular or vascular invasion. One NIFTP was reported after TT.

Among TIR3B patients undergoing HT, completion thyroidectomy was required in 4 (10.8%) patients because of histological finding of minimally or widely invasive follicular thyroid carcinoma (FTC) > 1  cm, whereas 2 (5.4%) patients presented occult PTMC with no need for further surgery according to current guidelines [10]. Among TIR3B patients undergoing TT, malignancy was found in 22 cases, mostly in the form of PTMC (15 out of 22 cases, 68.2%). Bilateral CND was performed in 2 patients, but no metastases were found at final histopathology. Extracapsular invasion was found in 2 patients and 1 patient presented vascular invasion. Two NIFTPs were reported.

Overall, the malignancy rate was 27.7%, with no significant differences between the two groups (17.2% in TIR3A patients vs. 31.1% in TIR3B patients, *p*=0.16). PTMC was the most frequently recorded type of cancer in both set of patients, with a total prevalence of 63.6% (21 out of 33 cases). A total of 47 HT were performed with no need for further operation in 43 (91.5%) patients.

A total of 91 (76.5%) patients underwent molecular testing on preoperative FNA cytologic samples. Of these, 48 patients were tested for both BRAF and RAS mutation, whereas 43 patients, who were operated between 2013 and 2015, were tested for BRAF mutation only, because—as stated before—the RAS test was not available at our institution until 2015. When considering BRAF testing alone, sensitivity was 29.6% (95% CI. 13.7%–50.2%) and specificity was 98.4% (95% CI. 91.6%–99.9%), with a positive predictive value (PPV) of 88.9% (95% CI. 51.2%–98.4%), a negative predictive value (NPV) of 76.8% (95% CI. 72.1%–80.9%), and an accuracy rate of 78.0% (95% CI. 68.1%–86.0%), respectively. When considering RAS testing alone, sensitivity was 43.7% (95% CI. 19.7%–70.1%) and specificity was 75.0% (95% CI. 56.6%–88.5%), with a PPV of 46.7% (95% CI. 27.9%–66.5%), a NPV of 72.7% (95% CI. 62.3%–81.1%), and an accuracy rate of 64.6% (95% CI. 49.5%–77.8%), respectively. Data are summarized in Tables 3 and 4.

## 4. Discussion

FNA cytology is currently regarded as the primary and most cost-effective diagnostic tool for evaluating thyroid nodules, being able to differentiate between benign and malignant disease in up to 70–80% of cases [10–13]. However, sensitivity drops when analyzing follicular-patterned neoplasms and further evaluations are required, including possibly unnecessary surgery and costly additional testing. Despite the efforts made to improve diagnostic sensitivity and accuracy of cytologic classifications, the indeterminate thyroid nodule remains a matter of debate among pathologists, especially when considering diagnostic criteria and clinical impact of molecular marker [14–18].

The present study aimed to assess the clinical impact of the 2014 SIAPEC classification on the management of indeterminate thyroid lesions, particularly with regard to malignancy rates, diagnostic impact of molecular markers, and relative care strategies. According to our results, the malignancy rate in TIR3B patients was nearly doubled when compared to that of TIR3A patients (31.1% vs. 17.2%, respectively). Although not significant on statistical analysis (*p*=0.16), this difference is in line with data from a recent meta-analysis, where cancer prevalence of TIR3A and TIR3B was 14% and 33%, respectively, considering NIFTP as a nonmalignant entity [37]. These data support the validity of the 2014 SIAPEC classification in cancer risk assessment.

As far as surgical treatment is considered, we tried to determine when surgery was indicated and what kind of surgical procedure was more appropriate (i.e., HT vs. TT). Based on the results of our study, less than one third (27.7%) of indeterminate nodules harbored a malignancy, and in nearly 64% of cases, the discovered thyroid cancer was unilateral, PTMC without aggressive histological features, extracapsular invasion, or lymph node involvement. In addition, the estimated need for further surgical treatment (i.e., completion thyroidectomy) was less than 10%. Therefore, HT could be considered as the treatment of choice for solitary TIR3B nodules without preoperative worrisome features, preventing overtreatment, and avoiding possible surgical complications [38–40].

On the other hand, it has to be considered that more than a half of TIR3B patients still received unnecessary surgical treatment for what was revealed to be a benign thyroid condition on final histological examination. Therefore, molecular testing has emerged as a powerful tool to improve the sensitivity and specificity of preoperative FNA cytology by enhancing risk stratification [33–35, 41]. In particular, up to 70% of thyroid cancers harbor at least one known genetic mutation and multiple markers have been studied, either alone or as a part of molecular panels. To date, the most common alteration in thyroid cancer is BRAF gene mutation, which most often occurs in papillary (40–45%) and poorly differentiated cancers (20–40%) and whose prevalence in indeterminate lesions varies between 15% and 40% [33, 42–44]. Another common alteration is RAS gene mutation, which is highly prevalent in follicular-patterned neoplasms, including FTCs, follicular variants of papillary thyroid carcinoma (40–50%), and benign follicular adenomas (up to 48%) [45–48].

In the present study, BRAF mutation in preoperative FNA cytologic samples was associated with high specificity (98.4%) and positive predictive values (PPV) (88.9%), although the sensitivity of the test was low (29.6%). These results are in line with the majority of literature studies, reporting a specificity of almost 100% with relative low sensitivity rates (15–62%) [42, 49–54]. We found only one false-positive mutation case, confirmed as adenomatous hyperplasia with underlying lymphocytic thyroiditis by surgical pathology. Surgically proven benign cases of false-positive BRAF V600E mutation have been documented in the literature [55–57]. False-positive results can be mainly explained by the trade-off of improved sensitivity by sacrificing specificity. Indeed, the use of more sensitive diagnostic methods (e.g., pyrosequencing and the polymerase chain reaction- (PCR-) based methods), detecting the BRAF mutation even in small amounts of mutant DNA, results in a false-positive rate of 0.08–5.4% (depending on the method used). Moreover, benign tumors can potentially share oncogenic mutation with their malignant counterparts. Thus, the real false-positive mutation rate of BRAF testing methods in FNAC specimens may be underestimated.

On the other side, RAS mutation presented low sensitivity and low PPV (43.7% and 46.7%, respectively) with mediocre specificity and negative predictive values (NPV) (75.0% and 72.7%, respectively). This is once more in accordance with literature data [58] and can be easily explained considering that RAS mutation can often be present also in follicular adenomas [45–48].

All things considered, our results corroborate the hypothesis that BRAF, with its high PPV for thyroid cancer, can be used to “rule-in” malignancy and guide treatment by referring patients with mutation-positive nodules to initial TT as opposed to diagnostic HT. Additionally, considering BRAF high specificity for malignancy, the absence of this mutation in TIR3A nodules (low-risk indeterminate) may strengthen the decision for nonoperative management, as outlined by current recommendations. As concerns RAS mutation, this analysis alone is unsuitable to predict malignancy in indeterminate thyroid nodules. However, it could be useful to “rule-out” malignancy and avoid unnecessary surgery in mutation-negative indeterminate nodules.

The present study has several limitations. Being a single-center experience based on retrospective nonrandomized analysis, the possibility of generalizing the results is potentially limited. In addition, the sample size is small, with a disproportion between TIR3A and TIR3B patients (24.4% and 75.6%, respectively). Moreover, we recorded a relatively high incidence of PTMCs, which are currently managed through more conservative treatments considering their indolent nature among all thyroid carcinomas [59, 60]. Last but not least, we did not consider the possibility of interoperator variability in the reading of preoperative FNA cytologic samples.

Despite these limitations, we demonstrated that the 2014 SIAPEC classification has improved diagnostic accuracy in patients with indeterminate thyroid nodules, especially with regard to malignancy rates and management strategies. Nevertheless, further studies are still required to avoid unnecessary surgical treatments. In this regard, molecular markers and immunochemistry may play a significant role in overcoming diagnostic limitations of basic cytological examination.

## 5. Conclusions

The differential diagnosis and proper treatment of indeterminate thyroid nodules remain a topic of great interest and discussion. The 2014 SIAPEC classification seems to have improved diagnostic accuracy and clinical management and its association with molecular markers may allow a better selection of surgical patients. However, further studies are required to reach a definitive agreement.

## Figures and Tables

**Table 1:** Comparison of the Bethesda, the RCPath, and the SIAPEC diagnostic categories.

BSRTC	RCPath	SIAPEC
I. Nondiagnostic or unsatisfactory	THY1/THY1c. Nondiagnostic /Cystic	TIR1. Nondiagnostic TIR1C : Nondiagnostic cystic

II. Benign	THY2/THY2c. Nonneoplastic	TIR2 : Nonmalignant /benign

III. Atypia of undetermined significance (AUS) or follicular lesion of undetermined significance (FLUS)	THY3a. Neoplasm possible. Atypical features present but not enough to place into any of the other categories	TIR3A. Low-risk indeterminate lesion

IV. Follicular neoplasm or suspicious for a follicular neoplasm	THY3f. Neoplasm possible—suggesting follicular neoplasm	TIR3B. High-risk indeterminate lesion

V. Suspicious for malignancy	THY4. Suspicious of malignancy	TIR4. Suspicious of malignancy

VI. Malignant	THY5. Malignant	TIR5. Malignant

**Table 2:** Surgical treatment, demographic, and pathologic data.

	TIR 3A (*n* *=* *29*)	TIR 3B (*n* = *90*)	*p* value
TIRADS score			
3, 4a, 4b (low-intermediate risk)	26	76	
Benign/malignant	22/4	57/19	
4c, 5 (high risk)	3^♦^	14^♦^	
Benign/malignant	2/1	5/9	

Nodule major diameter, mm (range)^*∗*^	23.4 ± 14 (4–60)	29 ± 18 (4–80)	

Type of surgery			
HT	10	37	
TT	19	53	

Reasons for TT			
Bilateral goiter	12	28	
Autoimmune thyroid disease	4	5	
Nodule growing in size	3	7	
BRAF/RAS mutation	9	12	
Prior head & neck irradiation	0	0	
Family history of thyroid cancer	0	1	

Completion thyroidectomy	0 (0%)	4 (10.8%)	0.56

Malignancy	5 (17.2%)	28 (31.1%)	0.16
pT1a	4	17¶	
pT1b	1	3	
pT2	0	5	
pT3	0	3	

Multifocality/TTs for cancer	1/4 (25%)	5/26 (19.2%)	Ns
Unilobar	0	0	
Bilobar	1	5	

Extrathyroid invasion	0	2 (7.1%)	Ns

Vascular invasion	0	1 (3.6%)	Ns

Aggressive variants	0	0	*—*

Lymph node metastases/lymph node dissections	1/1	0/2	*—*

Morphology			
Taller than wide	4	17	
Oval/round shape	25	73	

Margins			
Irregular	3	5	
Regular	26	85	

Microcalcifications			
Yes	5	12	
No	24	78	

Echogenicity			
Hypoechogen	6	44	
Nonhypoechogen	23	46	

Echostructure			
Solid	21	77	
Mixed	8	13	

*Note.* Data are presented as mean ± SD where applicable. TT, total thyroidectomy; HT, hemithyroidectomy; ns, nonsignificant. ^♦^0 cases of TIRADS 5 reported. ^*∗*^in bilateral goiter, size of the dominant nodule. ¶ one case of benignity of TIR3B lesion and coexistent occult PTMC in the controlateral lobe.

**Table 3:** BRAF status in TIR3 population.

	Number of cases	Benign	Malignant
BRAF−	82	63	19
BRAF+	9	1	8
	91	64	27

**Table 4:** RAS status in TIR3 population.

	Number of cases	Benign	Malignant
RAS−	33	24	9
RAS+ (9 NRAS Q61R mutations, 5 NRAS Q61K mutations, 1 KRAS G12A mutation)	15	8	7
	48	32	16

## Data Availability

Data were obtained from electronic database and manual search of studies on thyroid disease. The datasets used and analyzed during the current study is available from the corresponding author on reasonable request.
